# Metabolic diversity and co-occurrence of multiple *Ferrovum* species at an acid mine drainage site

**DOI:** 10.1186/s12866-020-01768-w

**Published:** 2020-05-18

**Authors:** Christen L. Grettenberger, Jeff R. Havig, Trinity L. Hamilton

**Affiliations:** 1 Department of Earth and Planetary Sciences, University of California, Davis, CA 95616 USA; 2 Department of Earth and Environmental Sciences, University of Minnesota, Minneapolis, MN 55455 USA; 3 Department of Plant and Microbial Biology, University of Minnesota, 218 Cargill Building, St. Paul, MN 55108 USA; 4 The BioTechnology Institute, University of Minnesota, St. Paul, MN 55108 USA

**Keywords:** *Ferrovum*, Carbon fixation, Iron oxidation, Metagenome, Acidophlic, Biofilm, Microcosm, Co-occurrence, Nitrogen, Denoised sequence variants

## Abstract

**Background:**

*Ferrovum* spp. are abundant in acid mine drainage sites globally where they play an important role in biogeochemical cycling. All known taxa in this genus are Fe(II) oxidizers. Thus, co-occurring members of the genus could be competitors within the same environment. However, we found multiple, co-occurring *Ferrovum* spp. in Cabin Branch, an acid mine drainage site in the Daniel Boone National Forest, KY.

**Results:**

Here we describe the distribution of *Ferrovum* spp. within the Cabin Branch communities and metagenome assembled genomes (MAGs) of two new *Ferrovum* spp. In contrast to previous studies, we recovered multiple 16S rRNA gene sequence variants suggesting the commonly used 97% cutoff may not be appropriate to differentiate *Ferrovum* spp. We also retrieved two nearly-complete *Ferrovum* spp. genomes from metagenomic data. The genomes of these taxa differ in several key ways relating to nutrient cycling, motility, and chemotaxis.

**Conclusions:**

Previously reported *Ferrovum* genomes are also diverse with respect to these categories suggesting that the genus *Ferrovum* contains substantial metabolic diversity. This diversity likely explains how the members of this genus successfully co-occur in Cabin Branch and why *Ferrovum* spp. are abundant across geochemical gradients.

## Background

Iron-oxidizing bacteria are common in mine-impacted water and acid mine drainage (AMD) environments which are typically characterized by low pH and high concentrations of dissolved metals [1–3]. Betaproteobacteria of the genus *Ferrovum* play important roles in biogeochemical cycling in AMD environments including carbon fixation and rapid oxidation of iron [4–9] and could be of value in bioremediation [10]. However, *Ferrovum* spp. are challenging and labor-intensive to culture and isolate because they often co-occur with heterotrophic *Acidiphilium* spp. or other Fe(II) oxidizers [7, 11, 12]. Indeed, *Ferrovum myxofaciens* strain P3 is the only *Ferrovum* spp. in culture to date, highlighting the value and need for characterization of this enigmatic genus via molecular techniques.

*Ferrovum* spp. occur in AMD environments with diverse geochemistry. *Ferrovum* spp. are abundant at sites with pH ranging from less than 3 to 7 [4–6, 13, 14] and with iron concentrations ranging from 2 μM [5] to 71 mM [7]. High-throughput sequencing and ‘omics techniques have aided in characterizing the metabolic potential of *Ferrovum* via non-culture-based techniques [15–18] across these large gradients of Fe and pH. These studies indicate that *Ferrovum* is a diverse genus composed of six clades (Groups I - VI) of closely related species and strains [18]. Still, only four *Ferrvoum* genomes are publicly available and these are from *Ferrovum* Groups I and IV. No genomes have been reported for Groups, II, III, or V. The published genomes and culture studies suggest that *Ferrovum* spp. are autotrophs that pair Fe(II) oxidation with carbon fixation [7, 15, 16, 18, 19]. *Ferrovum* spp. play important roles in nutrient cycling and some members of the genus also fix nitrogen [7, 19]. However, sequenced *Ferrovum* genomes have variable genomes especially with genes associated with motility, chemotaxis, biofilm formation, and nitrogen metabolism [15]. Furthermore, *Ferrovum* dominated communities exhibit morphological differences, including forming large streamers [5, 7, 14] or low extracellular polymeric substances [4] though it can be difficult to identify EPS in low pH environments [16]. These differences may help explain how *Ferrovum* is able to occupy a large range of pH and ferrous iron concentrations in AMD environments. However, the potential genetic and metabolic diversity within the genus is under-represented because there are no cultured representatives or genomes available for several *Ferrovum* clades.

In our previous studies of Cabin Branch, we reported the predominance of a single operational taxonomic unit (OTU; 97% similarity) in our 16S rRNA amplicon data most closely related to *F. myxofaciens* at this AMD site in the Daniel Boone National Forest in southern Kentucky. This taxon was abundant in diverse morphotypes including filament, floc, and ‘brain’ or ‘spongy’ mat biofilms over a pH range of 2.1 to 2.4 and Fe(II) concentrations of 448 to 5200 μmol/L [5]. Based on these data, we employed a cultivation-independent approach to characterize the genetic diversity and functional potential of this taxon using 16S rRNA amplicons and by examining the gene content of metagenome assembled genomes (MAGs) from the site.

## Results

### Geochemistry

**Fig. 1:**
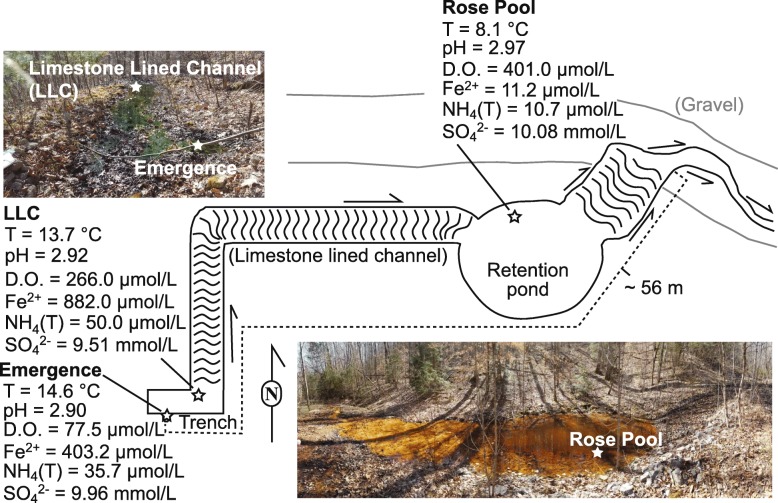
Sketch of the Cabin Branch sampling site with associated images and geochemistry. Emergence = point at which flow emerges from the groundwater source. Limestone lined channel (LLC) = fabricated channel lined with limestone gravels as part of remediation. Retention Pond (Rose Pool) = fabricated retention pond as part of remediation. Labeled stars denote sampling locations, with aqueous geochemistry shown for sites where metagenomic analyses were performed

All aqueous geochemistry data are reported in Table 1. pH values were acidic, ranging from 2.90 to 2.97 from the source to Rose Pool (Fig. 1). Temperatures were highest at the source (14.6 °C) and decreased down the outflow channel to 8.1 °C at the Rose Pool, consistent with warm groundwater cooling after emerging due to relatively cool ambient temperatures (~ 0 °C at the time of sampling). Conductivity increased from 940 μS/cm at the emergence to 2063 μS/cm at Rose Pool. Dissolved oxygen increased from a low of 77.5 μmol/L at the emergence sampling site to a high of 401 μmol/L (close to saturation) at Rose Pool. Anion concentrations in Cabin Branch were dominated by sulfate (9.51 to 10.08 mmol/L), with lower concentration of chloride (166 to 220 μmol/L). Cation concentrations were highest at the emergence and nearby first outflow sites, and lowest at the Rose Pool, including calcium (1.20 to 0.39 mmol/L), magnesium (0.69 to 0.20 mmol/L), potassium (0.18 to 0.03 mmol/L), and sodium (0.12 to 0.03 mmol/L).

**Table 1:** Geochemical and physical features for Cabin Branch samples

Location:		Emergence		LLC		Rose Pool	
Parameter	(Units)	Value	S.D.	Value	S.D.	Value	S.D.
pH		2.90		2.92		2.97	
mV		235		232		224	
Temperature	(°C)	14.6		13.7		8.1	
Conductivity	(μS/cm)	940		1853		2063	
D.O.	(μmol/L)	77.5		266		401	
Fe2+	(μmol/L)	403.2		882.0		11.16	
NH4(T)	(μmol/L)	35.7		50.0		10.7	
DIC	(mmol/L)	1.67		0.48		0.30	
δ13CVPDB	(‰)	−15.96	0.08	−13.03	0.08	−11.79	0.08
DOC	(μmol/L)	40.51	0.90	44.19	3.27	36.82	6.42
δ13CVPDB	(‰)	−22.50	1.53	−23.09	0.84	−23.95	0.29
Cl	(μmol/L)	175.58	1.10	220.04	61.99	166.13	0.44
SO4	(mmol/L)	9.96	0.16	9.51	0.14	10.08	0.06
P	(μmol/L)	9.55	0.49	4.49	0.35	bdl	
Na	(mmol/L)	0.12	0.00	0.12	0.03	0.03	0.00
K	(mmol/L)	0.17	0.00	0.18	0.04	0.03	0.00
Ca	(mmol/L)	1.121	0.028	1.198	0.256	0.390	0.007
Mg	(mmol/L)	0.666	0.012	0.686	0.156	0.201	0.001
Al	(μmol/L)	*1755*	*125*	1201	0.26	187.4	1.2
Si	(μmol/L)	1528	35	1454	66	nd	
Mn	(μmol/L)	76.84	0.63	43.19	0.13	40.23	0.28
FeTotal	(μmol/L)	*4684*	22	*4154*	*50*	249.6	1.8

Geochemical and physical measurements: meters/probes (pH, mV, Temperature,

Conductivity, Dissolved Oxygen (D.O.)), spectrophotometry (Fe(II),

NH4(T)), ion chromatography (Cl, SO4), IR-MS (dissolved inorganic carbon

(DIC), dissolved organic carbon (DOC), ICP-OES (P, Na, K, Ca, Mg, Si,

italisized values for Al and FeTotal), and ICP-MS (Al, Mn, Fe). LLC = limestone-

lined channel, S.D. = standard deviation, bdl = below detection limits,

Dissolved inorganic carbon (DIC, predominantly present as dissolved CO_2_ due to the low pH) concentration was highest at the emergence (1.67 mmol/L) and decreased down the outflow channel to 0.30 mmol/L at the Rose Pool. DIC δ^13^C values were more negative at the source (δ^13^C = − 15.96 ‰) and became more positive down the outflow channel with the most positive values at the Rose Pool (δ^13^C = − 11.79 ‰), consistent with preferential loss of ^12^CO_2_ through volatilization and uptake by autotrophs. Dissolved organic carbon concentration was consistently low at all sites (36.8 to 44.2 μmol/L) and had similar δ^13^C values (− 22.50 to − 23.95 ‰).

Potential nutrients measured included NH_4_(T), P, Mn, Fe(II), and Fe_(total)_. NH_4_(T) concentration was highest at the first outflow site (50.0 μmol/L) and lowest at Rose Pool (10.7 μmol/L). P concentration was highest at the emergence (9.55 μmol/L) and decreased to below detection limits at the Rose Pool. Mn concentration decreased from the highest value at the emergence (76.84 μmol/L) to the lowest at the Rose Pool (40.23 μmol/L). Total iron (Fe_total_) exhibited a similar trend as Mn and P, with the highest concentration at the emergence (4684 μmol/L) and the lowest at the Rose Pool in the outflow (249.6 μmol/L), though Fe(II) had concentrations significantly lower than Fe_total_ with the highest concentration at the outflow site downstream of the emergence (882 μmol/L) and the lowest at the Rose Pool (11.2 μmol/L).

### Inorganic carbon assimilation

Autotrophic incorporation of added ^13^CO_2_ was quantified in mesocosm experiments using treatments carried out under either light or dark (wrapped in aluminum foil) to characterize photoautotrophic and chemoautotrophic uptake, respectively. At the emergence, both green and white biomass was observed and autotrophic incorporation of ^13^CO_2_ was quantified in both biofilm types. Because we assumed the majority of uptake in the green biomass would be due to photoautotrophy and we could not control for oxygen production in this treatment, we performed one set of microcosms on the bulk biofilm in the light and then separated the biofilm types (green or white) for microcosms performed in the dark. In the light treatments of full biofilms (green and white biomass) C-uptake rates averaged 32.547 (± 7.949) and 21.691 (± 6.36) μg C uptake/g C_biomass_/hr. after one- and two-hour incubations, respectively. Dark treatments of the green biomass returned averages of 1.632 (± 0.454) μg C uptake/g C_biomass_/hr. after 1 hour and 0.746 (± 0.174) μg C uptake/g C_biomass_/hr. after 2 hours, while dark treatments of the white biomass returned averages of 0.305 (± 0.403) μg C uptake/g C_biomass_/hr. after 1 hour and 0.662 (± 0.240) μg C uptake/g C_biomass_/hr. after 2 hours. Biofilms with both green and white biomass together from the limestone-lined channel immediately downstream of the emergence returned average C-uptake rates of 35.947 (± 5.516) and 15.085 (± 0.106) μg C uptake/g C_biomass_/hr. for light treatments of one and two hours respectively and 2.586 (± 0.148) and 1.245 (± 0.035) μg C uptake/g C_biomass_/hr. for dark treatments after one and two hours. Rose Pool sediment incubations returned average C-uptake rates of 1.372 (± 0.061) μg C uptake/g C_biomass_/hr. for light treatments of 2 hours and 0.066 (± 0.132) μg C uptake/g C_biomass_/hr. for dark treatments of 2 hours. All C-uptake results are reported in Supplementary Table S1.

#### 16S rRNA

**Fig. 2:**
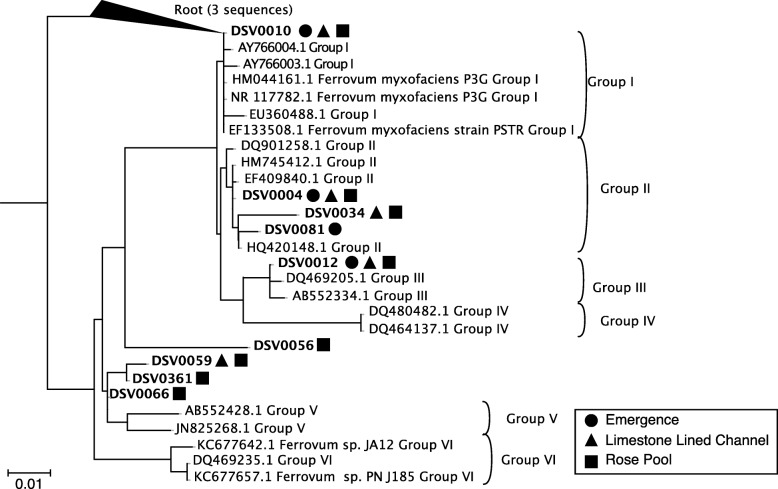
16S rRNA gene tree of *Ferrovum* spp. Group nomenclature from [18]. DSVs found in the emergence are indicated by circles, triangles indicate those found in the limestone lined channel, and squares those found in the Rose Pool

**Fig. 3:**
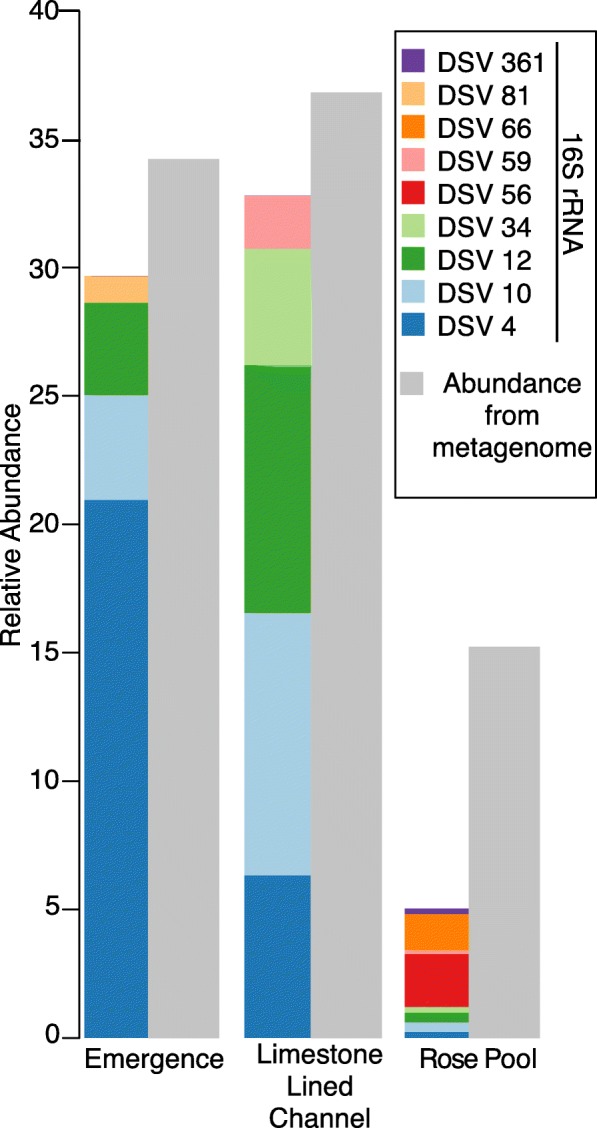
Relative abundance of* Ferrovum *DSVs in each of the sampled communities as determined by 16S rRNA sequencing. Each Ferrovum DSV is represented by a unique color. Grey bars indicate estimated abundance of Ferrovum from the metagenomic datasets

Thirty two thousand one hundred twenty four quality-controlled sequences were retrieved from the emergence, 41,988 from the limestone-lined channel, and 37,546 from the Rose Pool. These sequences were affiliated with 82, 276, and 286 denoised sequence variants (DSVs) respectively. Cabin Branch contains nine DSVs closely related to *Ferrovum myxofaciens* (Fig. 2). Four DSVs are present in the emergence sample, five in the limestone lined channel, and eight in Rose Pool (Figs. 1 and 2). DSVs affiliated with *Ferrovum* are most abundant in the limestone lined channel where they compose 32.8% of the community and least abundant in the Rose Pool where they compose 5.0% of the community. *Ferrovum* DSVs compose 29.6% of the emergence community. The abundance of each DSV varies between samples. DSVs 4, 10, 12, and 81 are the most abundant *Ferrovum* DSVs in the Emergence community, 4, 10, 12, 34, and 59 in the limestone lined channel, and DSVs 56 and 81 in the Rose Pool (Fig. 3). The DSVs are phylogenetically placed within *Ferrovum* groups I, II, III and between Groups IV and V (Fig. 2).

### Metagenomics

**Fig. 4:**
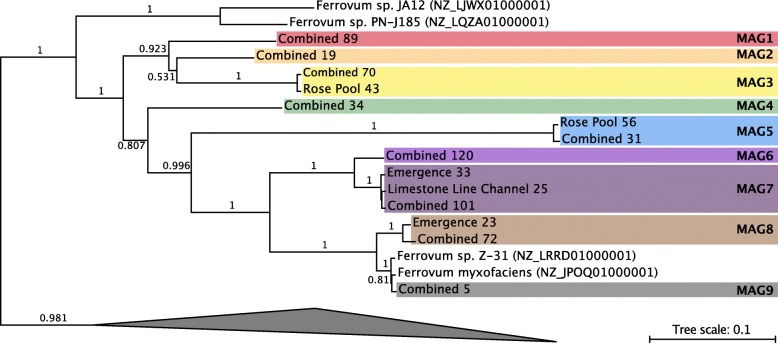
Concatenated gene tree containing all *Ferrovum* bins retrieved from the metagenomic datasets. Colored rectangles indicate the bins that belong to a given MAG. Bootstrap values (based on 100 bootstrap samplings) are shown for each node where bootstrap support is > 50%

74,021,852 to 101,886,314 metagenomic reads were retrieved per sample with an average GC content of 55% (Supplementary Table S2). Fourteen bins were retrieved that were phylogenetically placed within the genus *Ferrovum*. The resulting MAGs ranged from low- to high-quality drafts based on quality and contamination, but lack 16S rRNA gene sequences [20]. Five of these bins were retrieved from individually assembled metagenomes and 9 from the combined assembly. Based on average nucleotide identity (ANI), each of the *Ferrovum* bins from the individual assemblies was also present in the combined assembly and one bin was represented in the emergence, terrace, and combined assemblies (Supplementary Table S3). For MAGs that were present in more than one assembly, the highest quality bin from that MAG was used for further analysis (Table 2). Nine unique *Ferrovum* MAGs were present in the dataset. These MAGs shared 68.5 to 88.5% ANI with each other and between 68.6 and 99.4% ANI with published *Ferrovum* genomes (Supplementary Table S3). Based on a concatenated tree of ribosomal protein genes, eight of the nine MAGs occupy the phylogenetic space between the Group I *Ferrovum* sp. JA12 and sp. PN-J185 and the Group IV *Ferrovum* sp. Z-31 and the type strain (Fig. 4). Two MAGs, MAG-4 and MAG-7 are > 85% complete and have limited contamination. Therefore, we have described them in more detail below.

**Table 2:**
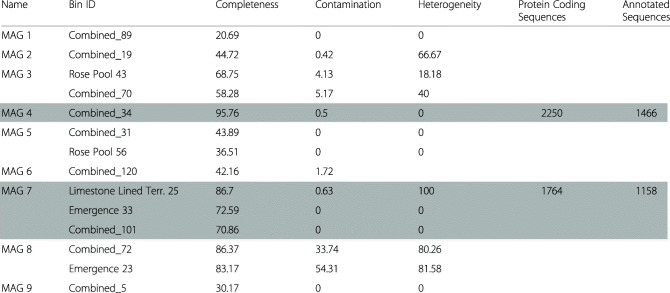
Metagenome assembled genomes. When a taxon was found in multiple assemblies, the MAG with the highest completeness and lowest contamination was used. The MAGs used for further analysis are shown in black text whereas those not used are shown in grey text. The MAGs used in the comparative analysis are highlighted by in grey

### MAG-4

MAG-4 is represented by a single bin which was retrieved from the combined assembly and is the most complete MAG recovered in this study. It is 95.7% complete with 0.4% contamination. MAG-4 contains 2250 gene coding sequences, 1466 of which were annotated by GhostKoala (Table 2). MAG-4 shares 69–74% ANI with the other MAGs and the published *Ferrovum* genomes (Supplementary Table S3). The MAG is phylogenetically positioned between the Group I *Ferrovum* spp. JA12 and PN-J185 and the Group VI type strain and sp. Z-31 (Fig. 4)

### Energy metabolism

**Fig. 5:**
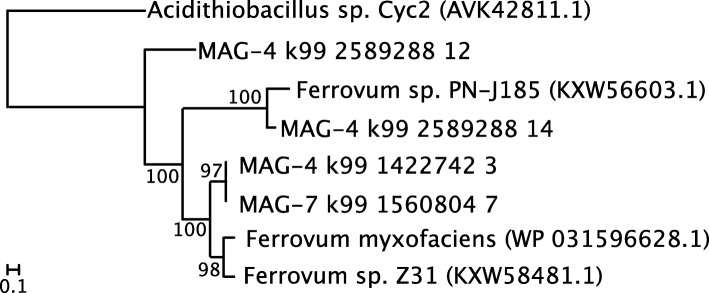
Maximum likelihood phylogeny phylogeny of Cyc-2 like gene found in published *Ferrovum* genomes and the MAGs retrieved in this study. Accession numbers are provided in parentheses. Numbers represent bootstrap support values based on 100 bootstrap samplings

**Fig. 6:**
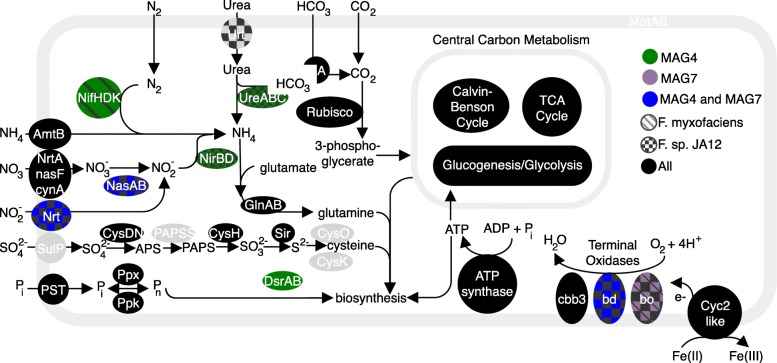
Potential cycling of C, S, P, and N in Ferrovum MAGs from Cabin Branch as predicted by the gene content of the MAGs. Proteins are color coded based on their presence or absence in each genome. Modeled after [18]

MAG-4 contains three homologs of the high molecular weight, Cyc-2-like protein previously found in *Ferrovum* spp. (Figs. 5 and 6). This protein is thought to facilitate the oxidation of Fe(II) to Fe(III) [16]. The MAG also contains homologs of the genes necessary for oxidative phosphorylation using a B/A type NADH dehyrodgenase (*nuoA-nuoN*) and succinate dehydrogenase (*sdhA-D*), a E/B/A type cytochrome *c* reductase, *cbb*_3_ and *bd* type terminal oxidases, and an F-type ATPase (Fig. 6).

#### Carbohydrate metabolism

MAG-4 encodes the enzymes necessary to fix carbon via the Calvin-Benson cycle except the enzyme needed to form ribose 5-phosphate from sedoheptulose-7 phosphate. The MAG also contains the genes for a nearly complete TCA cycle. It contains a nearly complete suite of genes for glycolysis.

#### Nutrient acquisition

MAG-4 encodes the structural proteins necessary for nitrogen fixation (NifHDK) and denitrification (NasAB, NirBD), but lacks the genes necessary to import inorganic nitrogen into the cell (Fig.  6). The MAG contains many of the genes necessary for assimilatory sulfate reduction but is missing PAPSS which reduces ammonium persulfate (APS) to 3-phosphoadenosine-5-phosphosulfate (PAPS). It also contains homologs of DsrA and DsrB which can be used to reduce sulfite to sulfide or to oxidize sulfide to sulfite (Fig. 6). The MAG also contains the genes necessary to import inorganic phosphate and use it in biosynthetic pathways (*PST, ppx, ppk*).

#### Motility

MAG-4 contains all the genes necessary to construct flagella except *motXY* which encode proteins are involved in flagellar rotation and *fliJT*. The MAG also contains the genes necessary for chemotaxis (*cheABCD*, *cheR*, and *cheVWZ*)

### MAG-7

MAG-7 is represented by bins retrieved from the emergence, limestone lined channel, and co-assembled metagenomes (Table 2). The highest quality bin is from the limestone lined terrace and is 86.7% complete with 0.63% contamination. The other two bins are 72.6 and 70.9% complete with 0% contamination. Because the most complete bin is only 86.7% complete, when genes were absent from that bin, we searched the other two less complete bins

#### Energy metabolism

MAG-7 contains one homolog to the high-molecular weight Cyc2 type protein found in other *Ferrovum* taxa (Figs. 5 and 6). The Cyc2 sequence is phylogenetically placed as a sister group to those from the type strain and *Ferrovum* sp. Z31 (Fig. 4). MAG-7 also contains homologs of the genes necessary for oxidative phosphorylation with a B/A type NADH dehydrogenase (*nuoA-nuoN*) and succinate dehydrogenase (*sdhA-D*), an E/B/A type cytochrome *c* reductase, *bo*, *bd*, and *cbb*_3_ type terminal oxidases, and an F-type ATP-ase.

#### Nutrient acquisition

MAG-7 encodes the genes necessary for reducing nitrate to nitrite (*nasAB*), but not those to further reduce the nitrate to ammonia (*nirBD*; Fig. 6). The MAG also contains the genes necessary to import inorganic phosphate and use it in biosynthetic pathways (*PST, ppx, ppk*). MAG-7 does not encode protein necessary for nitrogen fixation and lacks the genes necessary to transform urea to ammonia.

#### Carbohydrate metabolism

MAG-7 contains most of the genes necessary for carbon fixation via the Calvin-Benson cycle but like MAG-4 does not encode the enzyme needed to form ribose 5-phosphate from sedoheptulose-7 phosphate. The MAG encodes the proteins necessary for a complete TCA cycle. It contains a nearly complete suite of genes for glycolysis.

#### Motility

MAG-7 contains all the genes necessary to synthesize a functional flagellum except *motX* and *motY* which are also missing from MAG-4. The MAG also contains genes involved in bacterial chemotaxis including *mcp*, *aer*, *tar, cheABCD*, *cheR*, and *cheVY*, but it does not appear to contain *cheW* and *cheZ* which are present in MAG-4.

## Discussion

### Co-occurrence of *Ferrovum* spp. in cabin branch communities

All known *Ferrovum* spp. are obligate, Fe (II) oxidizing autotrophs that use O_2_ as a terminal electron acceptor [7, 16, 17]. Therefore, co-occurring *Ferrovum* spp. would presumably compete for common resources if they exploited identical resources, possibly including Fe (II) and oxygen, essential nutrients (e.g., N and P), and trace elements. At Cabin Branch, Fe (II) is present in mmol/L amounts constantly supplied by mine-impacted sources and is unlikely to be limiting. Dissolved oxygen is also present although the concentration is lower at the emergence and increases downstream. Ammonia and phosphate were detected at all sites. Alternately, gene content differences could explain co-existence of *Ferrovum* populations at Cabin Branch. Co-occurring strains of the same species have been found in a variety of environments including cyanobacterial blooms [21], and solar saltern/crystallization ponds [22, 23]. In some communities, successful co-occurrence results from genomic variation or differential gene expression between the strains of the co-occurring species. For example, six co-occurring strains of the Epsilonproteobacteria *Lebetimonas* acquired new functional genes via lateral gene transfer in sea mounts in the Mariana Arc [24]. Similarly, strains of *Salinabacter ruber* contain hypervariable regions in their genome which are associated with differences in surface properties [22]. Co-occurring strains of *S. ruber* also express different metabolite pools [23]. These differences likely allow co-occurring strains to avoid competing within the environment [22, 24]. Like these communities, the *Ferrovum* spp*.* at Cabin Branch may be able to co-exist due to variations in their genomes or in gene expression [7, 16, 17].

**Fig. 7:**
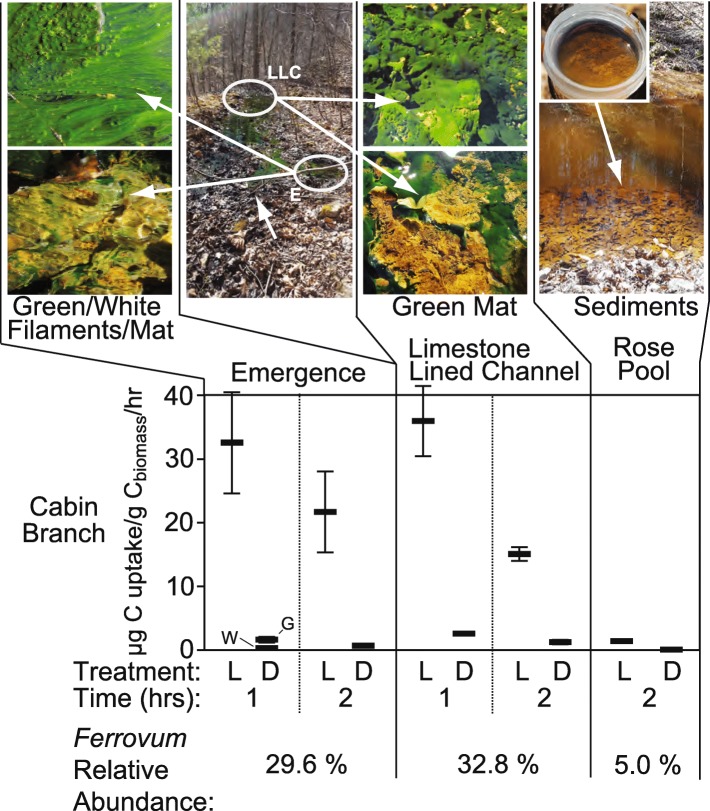
Images and carbon uptake rates for Cabin Branch emergence (E), limestone lined channel (LLC), and Rose Pool retention pond sites. L = light treatment, D = dark treatment (wrapped in aluminum foil), W = white filaments/mat, G = green filaments/mat

*Ferrovum* spp. compose over 25% of the bacterial community in the emergence and limestone lined channel communities and 5% of the Rose Pool community, based on the 16S rRNA amplicon libraries. The relative abundance of *Ferrovum* spp. appears to increase with the rate of non-photosynthetic inorganic carbon assimilation (Fig. 7). These data suggest that these populations are important primary producers in the environment because *Ferrovum* spp. are the only abundant chemoautotroph and therefore, likely the main contributor to dark carbon fixation. Our previous work on samples collected in 2015 reported a single *Ferrovum* OTU in Cabin Branch. However, that analysis relied on a definition of 97% identity in 16S rRNA sequences [5] which is the common cutoff for delineating species based on 16S rRNA gene sequence identity level, but likely underestimates bacterial diversity [25]. Currently, methods using unique sequences are becoming more common and may be more robust [26, 27]. Using a DSV approach on samples collected in 2017 as part of this research, we recovered nine unique *Ferrovum* taxa: four *Ferrovum* DSVs co-occur in the emergence community, five in the limestone lined channel, and eight in the Rose Pool sediments (Figs. 2 and 3). Genome-based studies using average nucleotide identity (ANI) and multilocus phylogenetic analysis are more robust than 16S rRNA-based analyses and are the best practice for assigning species affiliations in genomes [28]. Metagenomic data from Cabin Branch support the presence of multiple *Ferrovum* spp. at each site, suggesting that the diversity of *Ferrovum* spp*.* at AMD sites globally may be underestimated because studies have traditionally used an OTU approach [4, 5].

To co-occur successfully, the strains may partition the niche to use different resources as is seen in closely-related taxa or intraspecific competitors in eukaryotic systems [29–31]. If phylogenetic distance correlates with metabolic diversity, we might expect co-occurring strains of *Ferrovum* would be distantly related. Indeed, those that co-occur in the Cabin Branch communities span the Group I, II, and III *Ferrovum* clades and the limestone lined channel and Rose Pool also include DSVs that are not placed within a clade (Fig. 1). However, some co-occurring *Ferrovum* spp*.* are closely related to one another. Multiple DSVs from the same *Ferrovum* group are found in the same microbial community. For example, two Group II DSVs are found in all three communities sampled and the Rose Pool community contains three closely related DSVs phylogenetically placed basally to the Group V *Ferrovum* (Fig. 2). Therefore, phylogenetic distance may not determine whether taxa can coexist. However, published *Ferrovum* taxa vary in their gene content with respect to genes associated with motility and nitrogen cycling among others, likely due to lateral gene transfer [15, 16, 18]. Therefore, we can examine how genome content may allow co-occurring taxa to partition resources and avoid competition.

### Relating two new *Ferrovum spp.* (MAG-4 and MAG-7) to published species

Here, we compared our *Ferrovum* MAGs to two published *Ferrovum* species from *Ferrovum* Groups I (*F. myxofaciens* sp. P3G) and IV (*Ferrovum* sp. JA12) to explore if genomic variability could contribute to the co-occurrence of these taxa. Our two *Ferrovum* MAGs and the Group I and Group IV genomes share numerous features, but differ in key ways relating to energy metabolism, nitrogen cycling, and motility. Because the MAGs are incomplete, we only consider a feature to be missing if all of genes that code for key enzymes related to that feature are absent from the MAG.

#### Energy metabolism

All cultured *Ferrovum* spp. can grow autotrophically via iron oxidation [7, 15, 16, 18, 19] and we predict that the two MAGs presented here share that ability. Each genome contains high molecular weight, Cyc2-like proteins found in other *Ferrovum* taxa [16]. In the acidophilic Fe oxidizers *Acidithiobacillus ferrooxidans* and a *Leptospirillum* spp. Cyc2 is an Fe oxidase [32, 33]. Cyc2-like proteins are also highly expressed in neutrophilic Fe(II) oxidizers [33–35]. Therefore, this protein likely plays a role in Fe(II) oxidation in *Ferrovum*. In *Acidithiobacillus ferrooxidans* and *Gallionella ferruginea*, Fe(II) oxidation is paired with carbon fixation. The MAGs encode a nearly full suite of genes for carbon fixation via the CBB pathway but differ in the types of terminal oxidases they use for oxidative phosphorylation (Fig. 5). MAG-7, *F. myxofaciens* sp. P3G, and *Ferrovum* sp. JA12 all contain cytochrome *bo*_3_ type oxidases, but MAG-4 does not contain this type of cytochrome. Similarly, MAGs 4 and 7 and *Ferrovum* sp. JA12 contain a cytochrome *bd*-type quinol oxidase which is not present in *F. myxofaciens* sp. P3G (Fig. 5). Terminal oxidases differ in their affinity for oxygen and are differentially expressed depending on the concentration of oxygen [34]. For example, *E. coli* express cytochrome *bo*_3_ under oxic conditions, and cytochrome *bd*, which has a higher affinity for O_2_*,* under oxygen limiting conditions [34]. MAG-4 lacks genes for cytochrome *bo*_3_ which may indicate that this taxon does not colonize well-oxygenated environments that may be more suitable for MAG-7, *F. myxofaciens* sp. P3G, and *Ferrovum* sp*.* JA12. Similarly, MAGs 4 and 7 and *Ferrovum* sp*.* JA12 may be able to colonize microaerobic environments that are not ideal for *F. myxofaciens* sp*.* P3G which lacks high affinity terminal oxidases. In Cabin Branch, these differences may lead to MAGs 4 and 7 preferentially inhabiting different portions of the biofilm with MAG-7 inhabiting the better oxygenated surface of the biofilms and MAG-4 residing in more microaerobic niches within the biofilm.

#### Nitrogen cycling

*Ferrovum myxofaciens* sp. P3G can fix nitrogen, but this trait is not conserved within the genus. Neither *Ferrovum* sp. Z-31 nor PN-J185 are capable of nitrogen fixation [15, 18]. At Cabin Branch, MAG-4 encodes the enzymes necessary for N-fixation (NifH, D, and K) and the hydrolysis of urea (Fig. 6) while MAG-7 does not. The ability to fix nitrogen would allow MAG-4 to colonize low NH_4_ environments. Additionally, it may influence where MAG-4 resides in the biofilm community as nitrogenase proteins are oxygen sensitive [36]. Therefore, MAG-4 may preferentially reside in low-oxygen environments that provide enough oxygen to perform Fe(II) oxidation, but at low enough concentrations to protect nitrogenase from oxidative damage. This idea is supported by the presence of *cbb*_3_ and *bd* type oxidases in MAG-4 that are preferentially expressed in microaerobic conditions as discussed above.

#### Motility

*Ferrovum* spp. vary in their ability to be motile. *Ferrovum* sp. JA-12 and the closely related *Ferrovum* sp. PN-J185 lack genes both for flagella and for chemotaxis whereas *F. myxofaciens* sp. P3G contains the genes necessary for a functional flagellum [9, 15–17, 19]. The MAGs from Cabin Branch are more similar to *F. myxofaciens* sp. P3G in this respect, because they encode the genes necessary for the synthesis of a functional flagellum. MAG-7 and *F. myxofaciens* sp. P3G also contain homologs of *tar*, a chemotaxis related protein that responds to the presence of aspartate or maltose and *aer* which produces a protein that allow a chemotactic response to redox state [37, 38] potentially indicating that these taxa can respond to different environmental cues than MAG-4 which lacks these genes.

## Conclusions

We recovered co-occurring *Ferrovum* taxa with distinct metabolisms from the emergence and outflow of an AMD site. A DSV-based approach suggested multiple *Ferrovum* taxa co-occur which contrasts to previous OTU-based studies [5]. These data and the *Ferrovum* genomes recovered from metagenomic data highlight the limits of using OTU clustering approaches in identifying discrete species. We also identified the metabolic diversity among closely related *Ferrovum* spp. that likely facilities the co-occurrence of these taxa. Specifically, the differences in nutrient cycling, motility, and chemotaxis may facilitate co-occurrence without direct competition for resources. These physiological differences may also help to explain why *Ferrovum* spp. are nearly ubiquitous in AMD environments despite the geochemical diversity of these environments. These data help fill in gaps of missing diversity in the *Ferrovum* clade which are ubiquitous in AMD environments but difficult to culture. Finally, our data highlight genetic diversity necessary for closely related co-occurring species.

## Methods

### Site location and sampling

Cabin Branch is a site in the Daniel Boone National Forest in Kentucky near the border with Tennessee. Limestone was added to the channel as a passive remediation strategy and groundwater flows out from an emergence and across a limestone-lined channel before entering a pond, the Rose Pool (Figs. 1 and 7). The microbial communities within Cabin Branch are predominantly the Fe (II)-oxidizing taxon *Ferrovum myxofaciens* [5] Samples were collected on March 16, 2017 between 10 am and 2 pm. Sample collection was approved by and performed in collaboration with the staff at Daniel Boone National Forest.

### Geochemical analyses

#### Aqueous geochemistry

At the time of sample collection, temperature and pH were measured and recorded using a WTW 330i meter and probe (Xylem Analytics, Weilheim, Germany). A YSI 30 conductivity meter and probe (YSI Inc., Yellow Springs, OH, USA) was used to measure conductivity. Total ammonia (NH_4_(T) = NH_3_ + NH_4_^+^) and ferrous iron (Fe(II)) concentration were determined using Hach DR1900 Portable Spectrometers (Hach Company, Loveland, CO) using the salicylate method (Hach Method 8155) and the phenanthroline method (Hach Method 8146), respectively.

To examine water geochemistry including major ions, major cations, trace elements, and carbon concentration and isotopes, samples were collected and stored following previously described methods [39–41]. In brief, samples were collected in a 140-mL syringe, filtered through 0.8/0.2 μm polyethersulfone syringe filters, and dispensed into 15-mL polypropylene centrifuge tubes (for major anions), acid-washed 15-mL polypropylene centrifuge tubes (for major cations and trace elements), Labco Exetainers® (Labco Limited, Lampeter, UK) pre-flushed with He (for dissolved inorganic carbon (DIC)), and sterile 50-mL centrifuge tubes (for dissolved organic carbon (DOC)). Major anions were determined at the Analytical Geochemistry Laboratory in the Department of Earth and Environmental Sciences at the University of Minnesota using a Thermo Scientific Dionex ICS 5000+ ion chromatography system. Were stored in 15 mL polypropylene centrifuge tubes. Major cations and trace metals were determined at the Analytical Geochemistry Laboratory in the Department of Earth and Environmental Sciences using a Thermo Scientific iCAP 6000 series ICP-OES (inductively coupled plasma optical emission spectroscopy) or a Thermo Scientific X Series 2 ICP-MS (inductively coupled plasma mass spectrometry). DIC concentration and ^13^C isotopic signal analyses were performed on a GasBench II system interfaced to a Delta V Plus isotope ratio mass spectrometer (IR-MS) (Thermo Scientific, Bremen, Germany) by the Stable Isotope Facility (SIF) at the University of California, Davis for analysis. DOC concentration and ^13^C isotopic signal analyses were performed on a O.I. Analytical Model 1030 TOC Analyzer (O.I. Analytical, College Station, TX, USA) by the SIF at UC-Davis. The standard deviation for DOC was determined from replicate analyses of each sample. For each analysis, field blanks were taken using 18.2 MΩ/cm deionized water transported to the field in 1-l acid washed Nalgene bottles for comparison to samples.

Colorimetric tests are optimized for 20 °C and are known to have chemical interferences, thus the spectrophotometrically derived Fe(II) concentrations should be considered semi-quantitative whereas Fe_total_ is measured via ICP-MS or ICP-OES with samples that are acidified with concentrated HNO_3_ to keep metals in solution. Our assumption is that the majority of Fe_total_ is Fe(II) and most Fe(III) present precipitates immediately as Fe (OH)_3(S)_ and is collected on 0.2 μm filters during sample collection.

#### CO_2_ assimilation

In situ microcosms were performed to assess the potential for inorganic carbon uptake through the addition of NaH^13^CO_3_. Sample collection and the microcosm set-up followed previously described methods [39–41]. In summary, samples (biofilms, filaments, etc.) were place in sterile serum vials, overlaid with spring water, capped, and amended with NaH^13^CO_3_ (100 μM final concentration) (Cambridge Isotope Laboratories, Inc., Andover, MA, USA). All assays were performed in triplicate between 10 AM and 2 PM. To assess the potential for photoautotrophic NaH^13^CO_3_ uptake, microcosms were incubated in the light. In contrast, microcosms were wrapped in foil to assess the potential for chemoautotrophic (dark) NaH^13^CO_3_ uptake. At the emergence, both green and white filaments were present. We performed assays on each by sampling predominantly green filaments and predominantly white filaments. We assumed the majority of uptake in the green biomass would be due to photoautotrophy and thus performed one set of microcosms on the bulk filaments/biofilm (green + white) in the light and then separated the biofilm types (green or white) for microcosms performed in the dark. Following incubation, vials were flash frozen on dry ice and stored at − 80 °C until processed (described below). Calculation of assimilation rates is described below.

#### C and N concentration and stable isotope signals

Samples for ^13^C natural abundance were collected at the time of sampling using sterile spatulas. These were flash frozen on dry ice, stored in liquid N_2_ for transport and stored at − 80 °C until processed. To examine ^13^C signals in natural abundance samples and microcosms, samples were acidified (for microcosms), dried, and weighed following previously described methods [39–41]. Samples were analyzed via an Elementar pyrocube elemental analyzer (EA) periphery connected to an Isoprime 100 continuous flow IRMS (IR-MS) at the University of Minnesota. NIST Standard 2710 was used for linearity corrections. δ^13^C values were calibrated using reference standards USGS-40 and USGS-41 and checked with a laboratory working standard (glycine). The following precautions were taken to minimize cross contamination of natural abundance samples with samples that were amended with NaH^13^CO_3_: natural abundance samples were processed, weighed, and analyzed separate from labeled samples and all laboratory processing and weighing equipment was cleaned with 80% ethanol between each sample. In addition, to check for memory effects or cross contamination of samples, standard checks and blanks were included with each analysis batch (no memory effects or cross contamination were detected). Assimilation rates were calculated as described previously [39–41] using difference between the total amount of ^13^C in natural abundance samples and incubation assay replicates which represents total mass of ^13^C-labelled DIC in biomass after the incubation period (1–2 h). We performed a one-way ANOVA followed by post hoc pairwise comparisons within the R software package (R version 3.3.2) to compare mean ^13^C uptake rates and considered *p*-values < 0.05 as significantly different.

### Molecular analyses

#### DNA extraction

Triplicate samples for DNA extraction were collected at the time of sampling using sterile spatulas, flash frozen onsite and stored at − 80 °C until processed. DNA was extracted from each replicate sample (*n* = 3) using a DNeasy PowerSoil Kit (Qiagen, Carlsbad, CA, USA) according to the manufacturer’s instructions and quantified using a Qubit 3.0 Fluorometer (Invitrogen, Burlington, ON, Canada). Equal volumes of each extraction were pooled and submitted to the University of Minnesota Genomics Center (UMGC) for amplicon sequencing. As a negative DNA extraction control, we attempted to extract DNA from the filters used for the field blank water samples (described above). These extractions failed to yield detectable DNA and failed library preparation for amplicon sequencing (see below for amplicon sequencing details).

#### DNA sequencing

Amplicons were sequenced at the UMGC with MiSeq Illumina 2 × 300 bp chemistry targeting the V4 hypervariable region of bacterial and archaeal 16S SSU rRNA gene sequences (515F and 806r) [42] as described previously [40].

Preparation of amplicon libraries followed the UMGC’s improved protocol to detect which taxonomic groups that often go undetected using existing methods [43]. Each sample was sequenced once. Total DNA was submitted to the UMGC for metagenomic sequencing and sequenced using HiSeq2500 High-Output 2 × 125 bp chemistry. Three samples were sequenced / lane.

#### 16S rRNA analysis

16S rRNA sequences from Cabin Branch were used to examine community composition. Primers and unpaired sequences were removed using trimmomatic [44]]) The surviving reads were processed in dada2 (v.1.4) following the pipeline tutorial [27]. Briefly, forward reads were trimmed to 210 bp and reverse reads were trimmed to 120 bp based on their quality profiles. Sequences with ambiguous bases and those with more than 2 expected errors were removed. Error rates were estimated using the learnErrors command. Sequences were dereplicated using the derepFastq command and the unique sequence variates were inferred using the dada command. Forward and reverse reads were merged using mergePairs. Contigs shorter than 250 or longer than 256 bp and chimeric sequences were removed. The surviving unique, denoised sequences are referred to as denoised sequence variants (DSVs). Taxonomy was assigned using the Silva training set [45] Eukaryotic sequences and those unclassified at the domain level were removed. The closest cultured and environmental relatives were identified using BLASTN [46].

We retrieved 1589 aligned, nearly-full length *Ferrovum* sequences from the Silva non-redundant database to serve as a reference alignment for the analyses described below. A 16S rRNA phylogeny was constructed by retrieving the sequences used by Ullrich et al. [18] with *Ferritrophicum radicicola*, *Nitrospovibrio tenuis*, and *Nitrospira lenta* used as an outgroup. These sequences were aligned using MAFFT on the Cipres Science Gateway [47] using the aligned *Ferrovum* sequences from the Silva database as a reference alignment. A tree of the smaller subset of sequences was constructed in RAXML-HPC2 on XSEDE [48] also on the Cipres Science Gateway. Sequences of DSVs classified as *Ferrovum* were added to the alignment using the –addfrags option in MAFFT [49]. Non-full-length sequences were added to the tree using the evolutionary placement algorithm. Trees were rooted and visualized in the interactive tree of life [50]. Raw sequences were uploaded to the NCBI Sequence Read Archive under BioProject PRJNA554371.

#### Metagenomic analysis

Individual metagenomes were assembled and a co-assembly of all metagenomes was constructed following the “tutorial on assembly-based metagenomics” [51]].Trimmed, quality-controlled sequences were assembled using MegaHit [52] using default parameters except minimum contig length, which was set at 1000 base pairs. Reads were mapped to the assembly using bowtie2 [53] and depth was calculated using the jgi_summarize_bam_contig_depths command in Anvi’o [54]. Contigs were binned using default parameters in metabat using [55]. Bin completeness was determined with CheckM [56]. Protein coding regions were identified with prodigal (within CheckM) [57] and GhostKoala was used to annotate protein coding sequences [58]. Pathways were considered to be absent from the taxon when none of the key genes associated with that pathway were present in the MAG.

Each bin was uploaded to KBASE [59] and annotated with Prokka using the “Annotate Assembly and Re-annotate Genomes with Prokka (v1.12)” app. A concatenated gene tree containing each bin and four published *Ferrovum* spp. genomes was constructed using the “Insert Set of Genomes Into Species Tree 2.1.10” app. This app uses up to 49 ribosomal and single-copy genes to construct a phylogenetic tree. MAGs that were more closely related to *Ferrovum* spp. than other taxa were selected for further analysis. The pairwise ANI between each bin and published *Ferrovum* genomes was calculated using anvi-compute-ani in Anvi’o. If MAGs shared > 98% pairwise ANI and were phylogenetically cohesive, we considered them to be representing the same populations and the bin with the highest completeness was chosen for further analysis. Raw reads, assemblies, and MAGs were uploaded to the NCBI Sequence Read Archive under BioProject PRJNA554371. The abundance of Ferrovum in each metagenomes using One Codex [60].

##### 16S rRNA

A custom blast database containing the 16S rRNA gene from the *Ferrovum myxofaciens* type strain (NR_117782.1) using the makeblastdb command in BLAST+. Each of the *Ferrovum* bins were searched for 16S rRNA genes using BLAST and a cutoff value of E-30.

##### Cyc-2

A custom blast database of the high molecular weight Cyc-2 like proteins was constructed using the methods described above. Each of the *Ferrovum* bins were searched for the Cyc-2-like protein identified by Ullrich et al., [16] by blasting translated nucleotide sequences against the blast database with an E-value of E-120. The retrieved sequences, database sequences, and a sequence for Cyc-2 from an *Acidithiobacillus sp*. (AVK42811.1) were aligned using MAFFT and a maximum likelihood tree was constructed as described above. The tree was rooted on *Acidithiobacillus* and visualized in iTOL.

#### Comparison with other Ferrovum genomes

Existing *Ferrovum* genomes are located within either the Group I or Group IV *Ferrovum* clades [18]. The genome for *Ferrovum* sp. JA12 (Group I) and *Ferrovum myxofaciens* (Group IV) were downloaded from NCBI. CheckM was used to determine genome completeness, and within CheckM, prodigal was used to identify protein coding sequences. Ghost KOALA was used to annotate protein coding sequences using the genus_prokaryotes database. Protein coding sequences were also annotated with prokka. We then manually compared the gene content of MAGs to that of existing *Ferrovum* genomes.

## Supplementary information

**Additional file 1: Table S1.** Carbon uptake experiment results.

**Additional file 2: Table S2.** Sequencing and assembly statistics for metagenomes used in this research.

**Additional file 3: Table S3.** Average nucleotide identity of MAGs retrieved from the Cabin Branch metagenomes.

## Data Availability

All analyses tools used in the study are publicly available. 16S rRNA and functional genes of closely related species and outgroups were downloaded from NCBI databases and accession numbers are provided for these sequences. Raw reads and assembled scaffolds for the metagenomes and MAG sequences as well as the amplicon libraries have been deposited in the NCBI under BioProject PRJNA554371.

## Abbreviations

AMD: Acid mine drainage
ANI: Average nucleotide identity
APS: Ammonium persulfate 3-phosphoadenosine-5-phosphosulfate
DIC: Dissolved inorganic carbon
DSV: Denoised sequence variants
ICP-MS: Inductively coupled plasma mass spectrometry
ICP-OES: Inductively coupled plasma atomic emission spectroscopy
MAG: Metagenome assembled genomes
PAPS: 3-phosphoadenosine-5-phosphosulfate
TCA: Tricarboxylic acid cycle
