# Supplementary material for: Metabolic diversity and co-occurrence of multiple Ferrovum species at an acid mine drainage site
Source: BMC Microbiol. 2020 May 18;20:119. doi: 10.1186/s12866-020-01768-w (PMC7236192; doi:10.1186/s12866-020-01768-w)
Supplement: Supplementary file 1 — Additional file 1: Table S1. Carbon uptake experiment results. [file 12866_2020_1768_MOESM1_ESM.pdf]

Supplementary Table 1. Carbon uptake experiment results.

Site and Biofilm Description	Treatment	Time	Carbon uptake experiments	
			Average	S.D.
Emergence		(hrs)	(µg C uptake/g $C_{biomass}$/hr)	
Green/White Biofilm	Light	1	32.55	7.95
Green only	Dark	1	1.63	0.45
White only	Dark	1	0.31	0.40
Green/White Biofilm	Light	2	21.69	6.36
Green only	Dark	2	0.75	0.17
White only	Dark	2	0.66	0.24
Outflow 1				
Yellow/Green Biofilm	Light	1	35.95	5.52
Yellow/Green Biofilm	Dark	1	2.59	0.15
Yellow/Green Biofilm	Light	2	15.09	0.11
Yellow/Green Biofilm	Dark	2	1.25	0.03
Outflow 2				
Rose Pool Sediments	Light	2	1.37	0.06
Rose Pool Sediments	Dark	2	0.07	0.13

Average = average of three replicates, S.D. = standard deviation.
